# Differential Requirement of Beclin 1 for Regulating the Balance of Naïve and Activated CD4^+^ T Cells

**DOI:** 10.3389/fcell.2020.00834

**Published:** 2020-08-26

**Authors:** Rui Xia, Min Yang, Xiaorui Fu, Wenwen Du, Xin Gao, Gang Li, Sarangarajan Ranganathan, Xueguang Zhang, Jingting Jiang, Binfeng Lu

**Affiliations:** ^1^Department of Immunology, Institute of Medical Biotechnology, Soochow University, Suzhou, China; ^2^Department of Immunology, School of Medicine, University of Pittsburgh, Pittsburgh, PA, United States; ^3^Department of Oncology, The Second Affiliated Hospital of Soochow University, Soochow University, Suzhou, China; ^4^Department of Oncology, The Third Affiliated Hospital of Soochow University, Changzhou, China; ^5^Department of Oncology, The First Affiliated Hospital of Zhengzhou University, Zhengzhou, China; ^6^Department of Pathology, UPMC Children’s Hospital of Pittsburgh, Pittsburgh, PA, United States

**Keywords:** naive T cell, Beclin 1, effector T cell, colitis, autoimmune, cell death

## Abstract

Autophagy is highly regulated and plays a multitude of roles during T cell-mediated immune responses. It has been shown that autophagy deficiency in T cells results in a decrease in total T cells, including naïve T cells in young mice, but the mechanism is still not understood. Here, similar to what happened in young mice, we showed that T cell-specific deletion of *Beclin 1/Atg6* (*Becn1* −/−) resulted in decreases in the percentages of CD4^+^, CD8^+^, and regulatory T cells in adult mice. In addition, we found that the effector to naïve T cell ratio was increased in older mice. Also, as mice grew older, *Becn1* −/− mice progressively lost weight and developed severe colitis. Analysis of inflamed tissues demonstrated increases in the portion and cytokine production of effector T cells. In contrast, the TCR-transgenic *Becn1* −/− mice had similar numbers of naïve T cells compared to WT controls. Similar to bulk T cells, the TCR-transgenic *Becn1* −/− T cells generated much lower numbers of effector T cells compared to WT controls after activation *in vitro*. These data suggest that autophagy is not required for maintaining the naïve T cell but required for the generation of effector T cells *in vivo*.

## Introduction

Autophagy is a highly regulated cellular process during the life cycle of T cells. The numbers of autophagosomes are greatly increased upon T cell activation and are also regulated by cytokines (Li et al., 2006; Pua et al., 2007). Lack of nutrient and inhibition of mTOR can also induce autophagy in activated T cells (Li et al., 2006; Jia and He, 2011). It has been shown that autophagy deficient T cells undergo elevated levels of programmed cell death after activation (Pua et al., 2007; Kovacs et al., 2012). In addition, autophagy is required for the survival of effector CD8^+^ T cells during viral infection and autoreactive CD4^+^ T cells during the course of the experimental autoimmune encephalomyelitis (EAE) (Kovacs et al., 2012; Schlie et al., 2015). It has been shown that autophagy blockade in activated T cells resulted in greater levels of apoptotic proteins, leading to increased levels of apoptosis (Kovacs et al., 2012). Despite the evidence for a prosurvival role of autophagy in activated T cells, autophagy has also been shown to promote cell death in a murine T cell line and HIV-infected human CD4^+^ T cells (Espert et al., 2006; Li et al., 2006). The exact role of autophagy in T cell survival is therefore dependent on cellular context.

Naïve T cells, characteristically expressing low levels of CD44 and high levels of CD62L, are quiescent, long-lived, and slow-proliferating in immune-intact mice (Sprent and Tough, 1994; Clarke and Rudensky, 2000; Dorfman et al., 2000; Sprent et al., 2008). In mice, the vast majority of the naïve T cell pool is sustained by thymic exodus of recently developed T cells (den Braber et al., 2012). Upon recognition of environmental antigens, naïve T cells are activated, differentiate into effector and memory T cells, and change their surface phenotype to CD44^*high*^ and CD62L^*low*^. Thus, the number of naïve T cells is controlled by thymic output, survival of naïve T cells, and activation by MHC/peptide complexes. Autophagy deficiency in T cells results in severely reduced numbers of naïve CD4^+^ and CD8^+^ T cells in the secondary lymphoid organs, without affecting thymic T cell development (Pua et al., 2007; Kovacs et al., 2012; Parekh et al., 2013; Wei et al., 2016), suggesting a critical role of autophagy in regulating the number of naïve T cells (Pua et al., 2007; Kovacs et al., 2012; Parekh et al., 2013; Wei et al., 2016). The mechanisms by which autophagy regulates naïve T cells numbers are not understood.

In this study, we investigated the impact of autophagy blockade on naïve and effector/memory T cell populations in adult mice with a deletion of *Beclin 1* in all T cells (*Becn1* −/−). In order to further determine the role of autophagy in naïve T cells, we utilized a TCR transgenic system to prevent naïve T cell activation by environmental antigens. Our study helps to clarify the role of autophagy in homeostasis of naïve T cells and autoimmunity.

## Results

### Beclin 1 Deficiency in T Cells Led to Severe Reduction in the Percentage of Naïve T Cells, but Greatly Increased Percentages of Effector/Memory T Cells in Adult Mice

**FIGURE 1:**
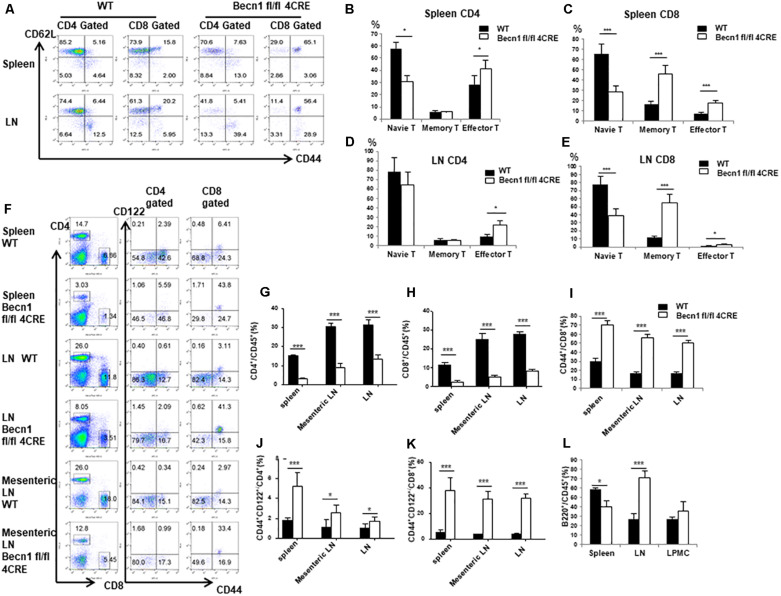
Autophagy blockade in T cells leads to systemic changes in T lymphocytes in secondary lymphoid organs. Lymphocytes were isolated from spleens and lymph nodes from 16-week-old WT and *Becn1* –/– mice. **(A)** Percentages of naïve (CD44^–^ CD62L^+^), central memory (CD44^+^ CD62L^+^), and effector (CD44^+^ CD62L^–^ ) T cells were analyzed by flow cytometry. **(B–E)** Statistical analysis of percentages of naïve, memory, and effector T cells depicted in panel **(A)**. **(F)** Flow cytometric analysis of percentages of CD4^+^ and CD8^+^ T cells (left) and their CD44^+^ CD122^+^ proportion (right) in spleens, lymph nodes, and mesenteric lymph nodes from WT and *Becn1* –/– mice. **(G–K)** Statistical analysis of percentages of T cell subsets depicted in panel **(F)**. **(L)** Percentage of B cells in spleens, lymph nodes, and lamina propria from WT and *Becn1* –/– mice. Data are representatives of three independent experiments. At least three control and *Becn1* –/– mice in each experiment. Bar charts represented mean of and error bars represented SEM. *P < 0.05, ***P < 0.001 by Student’s *t*-test.

Our previous studies have established that Beclin 1 deficiency in T cells resulted in reduction of naïve CD4^+^ and CD8^+^ T cells in young mice. We then further examined the long-term effect of Beclin 1 deficiency on total T cell population in adult mice. We observed a significant reduction of the percentage of CD44^*low*^ CD62L^*high*^ phenotype naïve T cells in both CD4^+^ and CD8^+^ T cells in the spleen and CD8^+^ T cells in the lymph node of *Becn1* −/− mice compared with WT mice (Figures 1A–C,E). We found an increase of the percentage of CD44^*high*^ CD62L^*low*^ effector memory T cells in both CD4^+^ and CD8^+^ T cells in spleens and lymph nodes of the *Becn1* −/− mice compared with WT mice (Figures 1A–E). In addition, we also observed increases in central memory CD8^+^ T cells in *Becn1* −/− mice compared to WT controls (Figures 1A–E). Despite the increase in memory/effector T cells, the percentages of CD4^+^ and CD8^+^ T cells were decreased in spleens and lymph nodes (Figures 1F–H). Consistent with the role of IL-15 in the expansion and homeostasis of memory T cells, we found an increase in CD44^*int*^ CD122^+^ CD4 and CD8 T cells in spleens, lymph nodes, and mesenteric lymph nodes of *Becn1* −/− mice compared with WT control mice (Figures 1F–L). Collectively, Beclin 1 deficiency in T cells resulted in decreases in the percentage of naïve T cells and increases in the percentage of effector and memory T cells in adult mice.

### Increased Percentages of T Cells Producing Effector Cytokines in *Becn1* −/− Mice

**FIGURE 2:**
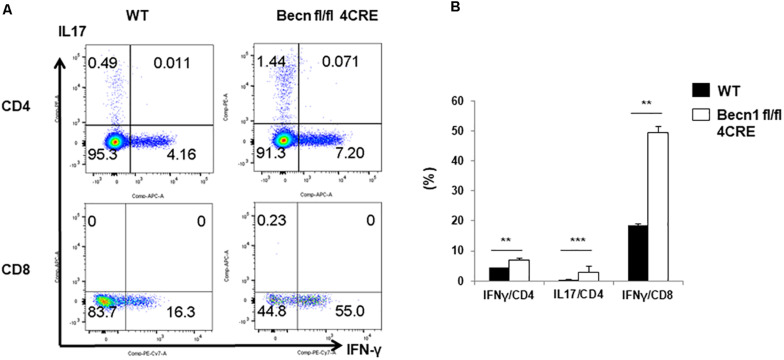
Cytokine production by peripheral CD4 and CD8 T cells. Lymphocytes were isolated from spleens of WT and *Becn1* –/– mice. **(A)** IFN-γ and IL-17 expression by CD4^+^ and CD8^+^ T cells were analyzed by flow cytometry. **(B)** Statistical analysis of panel **(A)**. Data are representatives of three independent experiments. Bar charts represented mean of and error bars represented SEM. **P < 0.01, ***P < 0.001 by Student’s *t*-test.

In order to further establish whether effector T cells were increased in *Becn1* −/− mice, we quantified IFN-γ and IL-17 producing CD4^+^ or CD8^+^ T cells *ex vivo* (Figures 2A,B). We found that the percentage of IFN-γ-producing CD4^+^ and CD8^+^ T cells and IL-17-producing CD4^+^ T cells were much higher in *Becn1* −/− mice than WT mice. These data suggested that active T cell-mediated immune or autoimmune responses were present in in *Becn1* −/− mice.

### Beclin 1 Deficiency Decreased the Percentage of Treg

**FIGURE 3:**
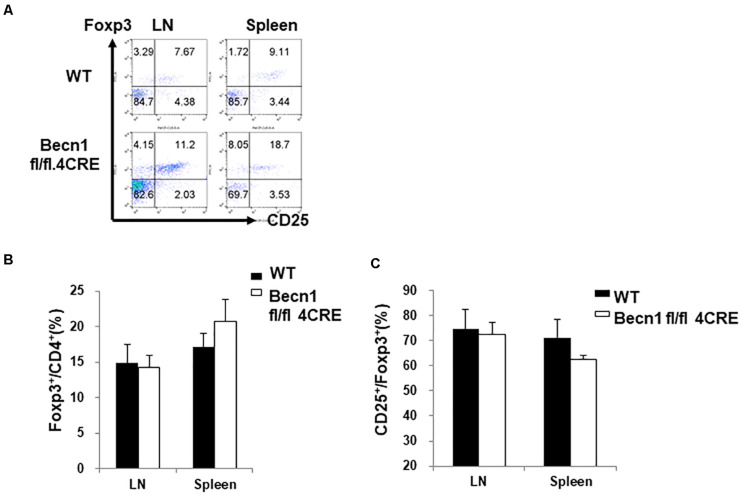
Lack of changes in the percentage of Treg in secondary lymphoid organs. Lymphocytes were isolated from spleens and lymph nodes of WT and *Becn1* –/– mice. **(A)** Flow cytometric analysis of Foxp3 and CD25 expression by CD4^+^ T cells. Statistical analysis of frequencies of CD4^+^ Foxp3^+^ **(B)** and CD25^+^ Foxp3^+^ **(C)** T cells. Data are representatives of three independent experiments. Three WT and *Becn1* –/– mice were used for each experiment. Bar charts represented mean of and error bars represented SEM.

We also examined the percentage of regulatory T cells within the CD4^+^ T cells. We found no significant difference in the percentage of Foxp3^+^ T cells within the CD4^+^ T cells (Figures 3A,B). There was also no significant difference in the CD25 expression in the Treg compartment (Figure 3C). Since there was a decrease in percentages of total CD4^+^ T cell numbers, the total percentage of Treg was also smaller in the *Becn1* −/− mice than in WT control mice.

### Increased Portions of Myeloid Cells in *Becn1* −/− Mice

**FIGURE 4:**
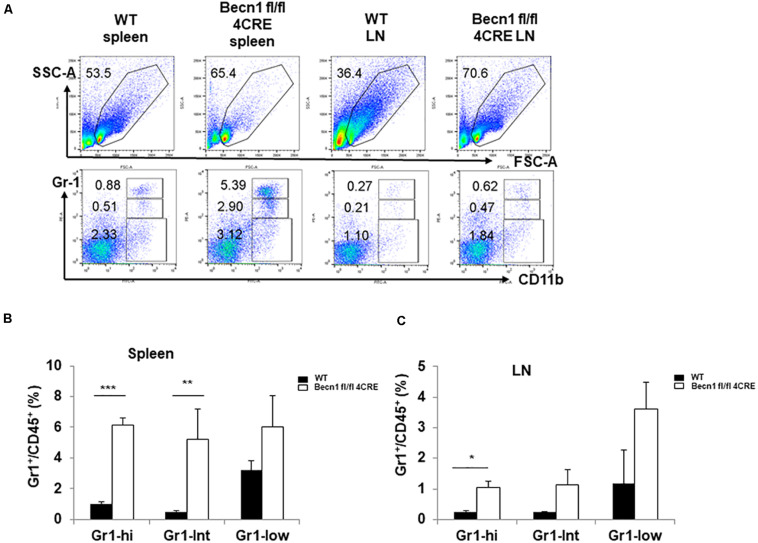
Autophagy blockade in T cells resulted in changes in innate cells. **(A)** The flow cytometric analysis of CD11b^+^ myeloid cells in spleens and lymph nodes from WT and *Becn1* –/– mice. **(B,C)** Statistical analysis of percentages of myeloid subsets in spleens and lymph nodes from WT and *Becn1* –/– mice. Data are representatives of three independent experiments. Three WT and *Becn1* –/– mice were used for each experiment. Bar charts represented mean of and error bars represented SEM. *P < 0.05, **P < 0.01, ***P < 0.001 by Student’s *t*-test. Five WT and *Becn1* –/– mice were used for each experiment. Bar charts represented mean of and error bars represented SEM. *P < 0.05 by Student’s *t*-test.

Chronic inflammation is usually associated with increases in myeloid derived suppressor cells (MDSC). We quantified the myeloid cells in the spleen. Our data showed a significant increase in the percentages of CD11b^+^ Gr-1^*high*^ MDSC and CD11b^+^ Gr-1^*int*^ MDSC in spleens and lymph nodes of *Becn1* −/− mice compared with WT mice (Figures 4A–C). The percentages of CD11b^+^ Gr-1^–^, which contains macrophages and dendritic cells, were not significantly changed between *Becn1* −/− mice and WT control mice. These findings were consistent with a chronic inflammatory condition in adult *Becn1* −/− mice.

### Beclin 1 Deficiency in T Cells Led to Severe Colitis in Adult Mice

**FIGURE 5:**
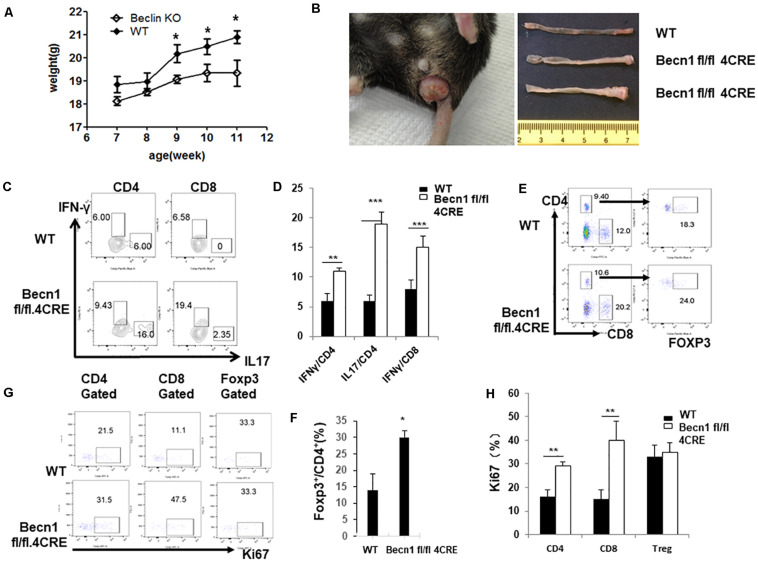
Autophagy-deficient mice developed spontaneous IBD. **(A)** Weight of WT and *Becn1* –/– mice over time (*N* = 8). **(B)** Gross morphology of prolapse from a 16-week-old *Becn1* –/– mouse and control mice (left) and colons from 16-week-old WT and *Becn1* –/– mice (right). **(C)** Lymphocytes were isolated from lamina propria of WT and *Becn1* –/– mice. IFN-γ and IL-17 expression by CD4^+^ and CD8^+^ T cells was analyzed by flow cytometry. **(D)** Statistical analysis of panel **(C)**. **(E)** Flow cytometric analysis of Foxp3 expression by CD4^+^ T cells. **(F)** Statistical analysis of percentages if Foxp3^+^ CD4^+^ T cells in WT and *Becn1* –/– mice. **(G)** Ki67 expression by CD4^+^, CD8^+^ T, and Treg cells were analyzed by flow cytometry. **(H)** Statistical analysis of panel **(G)**. Data are representatives of three independent experiments. Three WT and *Becn1* –/– mice were used for each experiment. Bar charts represented mean of and error bars represented SEM. **P < 0.01, ***P < 0.001 by Student’s *t*-test.

Defects in autophagy have been found by genetic association studies to confer susceptibility to several autoimmune and inflammatory disorders, particularly inflammatory bowel disease (Jones et al., 2013). We found that *Becn1* −/− mice started to have a lower body weight than WT mice 9 weeks after birth, and this weight loss became more significant as mice aged (Figure 5A). We also observed that about 70% of *Becn1* −/− mice (*N* > 50) showed rectal prolapse around 4 months after birth (Figure 5B). Additionally, we found that the colon of *Becn1* −/− mice were significantly elongated compared with WT mice (Figure 5B). This suggested that the *Becn1* −/− mice developed severe colitis. Therefore, Beclin 1 deficiency in T cells resulted in chronic inflammation of the colon.

Since *Becn1* −/− mice developed colitis, we characterized the lamina propria lymphocytes to examine the characteristics of T cells. We observed increased IFN-γ and IL-17 producing CD4^+^ and IFN-γ producing CD8^+^ T cells in *Becn1* −/− mice compared to WT mice (Figures 5C,D). We also found an increase in the percentage of Foxp3^+^ CD4^+^ T cells in the lamina propria (Figures 5E,F). We also measured the proliferation of T cells by examining Ki67 expression in CD4^+^ T cells, CD8^+^ T cells, and Treg. The proliferative rates were higher for CD4^+^ T cells and CD8^+^ T cells from the lamina propria of *Becn1* −/− mice when compared to WT mice (Figures 5G,H). In contrast, proliferation rates were similar between WT and *Becn1* −/− Treg in the lamina propria. These data suggest the tissue inflammation in *Becn1* −/− mice was driven by actively proliferating effector T cells.

### The TCR Transgene Prevented T Cell Loss and Colitis in *Becn1* −/− Mice

**FIGURE 6:**
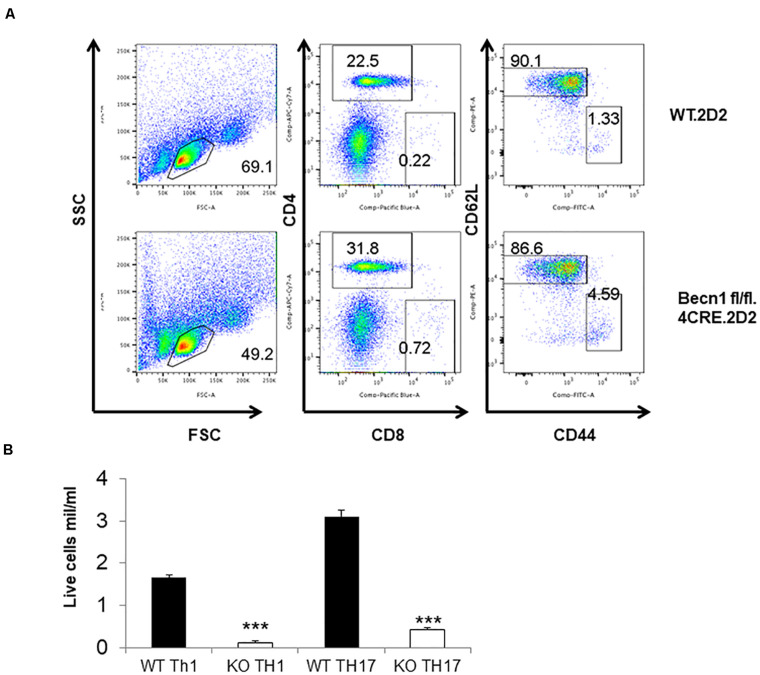
Expression of the TCR transgene normalized the number of CD4^+^ T cells in *Becn1* –/– mice. **(A)** Splenocytes from 2D2.WT and 2D2.*Becn1* –/– were subject to flow cytometric analysis. **(B)** Naïve CD4^+^ T cells from 2D2.WT and 2D2. *Becn1* –/– were cultured in the Th1 or Th17 conditions for 4 days. The number of live cells were determined. Bar charts represented mean of and error bars represented SEM. ***P < 0.001 by Student’s *t*-test.

In order to study the effect of Beclin 1 deficiency on naïve CD4 T cells, we bred 2D2.TCR transgene to *Becn1* −/− mice to generate 2D2.*Becn1* −/− mice. As expected, 99% T cells were CD4^+^ T cells in 2D2.WT and 2D2.*Becn1* −/− mice (Figure 6A). Interestingly, the number of CD4^+^ T cells was similar between 2D2.WT and 2D2.*Becn1* −/− mice (Figure 6A). Even around 4 months of age, about 90% of T cells are in the naïve state in both 2D2.WT and 2D2.*Becn1* −/− mice. No colitis was observed in 2D2.WT and 2D2.*Becn1* −/− mice for at least 8 months of age. In addition, IL2 production by naïve 2D2 WT and *Becn1* −/− CD4^+^ T cells was similar (data not shown). Despite the normalization of naïve T cell number *in vivo*, when activated and cultured *in vitro* for 72 h, 2D2.*Becn1* −/− T cells generated much fewer activated live T cells compared to 2D2.WT T cells (Figure 6B), suggesting Beclin 1 is required for survival of activated T cells.

## Discussion

The role of autophagy in T cell-mediated immune processes is complicated because of its differential involvement in many functions of T cells and various T cell subsets. Therefore, the exact function of autophagy in T cells needs to be studied in well-defined experimental systems. We found that T cell-specific deletion of *Becn1* resulted in systemic activation of T cells in adult mice, consistent with similar findings by other groups (Parekh et al., 2013; Kabat et al., 2016; Wei et al., 2016). We focused on the gastrointestinal (GI) tract and found strong evidence of colitis that is associated with an increase in the function and number of effector T cells. To further define the role of autophagy in naïve T cells, we generated TCR transgenic mice that had the *Becn1* deletion in T cells. We found that the reduction in the T cell number and systemic inflammation was prevented in the TCR transgenic *Becn1* −/− mice. Our results suggest that, unlike activated T cells, autophagy is not required for the survival of naïve T cells.

It has been suggested that reduction of naïve T cells in autophagy deficient mice is due to defects in naïve T cell homeostasis. Our data obtained using a TCR transgenic system, however, suggest that the development and homeostasis of naïve T cells is normal in T cell-specific autophagy deficient mice. We would like to propose a different explanation for the reduction of total T cell number in *Becn1* −/− mice, which is likely due to the combined effect of Treg deficiency and increased activation of CD4^+^ and CD8^+^ T cells. Our new model can reconcile all the published data. It has been reported that the number and functional integrity of Treg is reduced when autophagy is defective (Parekh et al., 2013; Kabat et al., 2016; Marcel and Sarin, 2016; Wei et al., 2016). The deficiency of Treg can result in increased activation of naïve T cells after encountering environmental antigens and self-antigens. Once *Becn1* −/− T cells are activated, they become prone to cell death (Pua et al., 2007; Kovacs et al., 2012). Thus, the increased activation potential of naïve T cells and susceptibility to activation induced cell death in combination lead to the reduction of the number of naïve T cell in the *Becn1* −/− mice.

Autophagy genes, such as ATG16L1, have been associated with inflammatory bowel disease (IBD), particularly Crohn’s disease (CD) (Hampe et al., 2007; Parkes et al., 2007; Rioux et al., 2007). Dysregulation of both canonical and non-canonical autophagy pathways in Paneth cells, macrophages, and dendritic cells has been shown to contribute to colitis (Saitoh et al., 2008; Cadwell et al., 2010; Chu et al., 2016). Our study suggests that autophagy deficiency in T cell can also potentially contributes to IBD. Although we have established that Beclin 1 plays a crucial role in autophagy in T cells (Kovacs et al., 2012), Beclin 1 has also been shown to have autophagy-independent functions (Kang et al., 2011; Wirawan et al., 2012), which warrants further examination in the future.

Several factors might have collectively contributed to the hyper inflammatory status in adult *Becn1* −/− mice. First, Beclin 1 is required to maintain the number of Treg. Second, Beclin 1 is required for the survival of activated T cells. Therefore, after activation, a majority of T cells undergo apoptosis. This leads to a reduction of total T cells. Third, the resulting lymphopenia allows increased proliferation of effector T cells, which makes large amounts of inflammatory cytokines. Fourth, it is possible that autophagy is differentially required for the function, proliferation, and survival of effector versus central memory T cells. Autophagy blockade might lead to the generation of more effector T cells. This point needs to be carefully examined in the future using appropriate experimental systems.

## Materials and Methods

### Mice

C57BL/6 mice were purchased from the Jackson Laboratory. The T cell-specific Beclin 1 deficient (*Becn1* −/−) mice on the C57BL/6 background were generated as described (Kovacs et al., 2012). 2D2 TCR transgenic mice (Bettelli et al., 2003) were bred with *Becn1* −/− mice and then intercrossed to generate 2D2.*Becn1* −/− mice. All mice were maintained under specific pathogen-free conditions. The Institution Animal Care and Use Committee at University of Pittsburgh has approved all animal work.

### Processing of Tissues

Spleens were isolated and mashed completely in Hank’s buffer (containing 1% FBS), cells were then treated with ACK lysis buffer and filtered through a 50-μm cell strainer to obtain single cell suspension. Inguinal lymph nodes, axillary lymph nodes, and mesenteric lymph nodes were isolated and mashed completely in Hank’s buffer (containing 1% FBS) and then filtered through a 50-μm cell strainer to obtain single cell suspension. For isolation of lamina propria cells, intestines were harvested and Peyer’s patches were removed, the intestines were opened longitudinally and the tissues were then minced and incubated in HBSS containing 5 mM EDTA and 1 mM DTT at 37°C for 20 min to remove epithelial cells, villus cells, subepithelial cells, and IELs, which was followed by digestion in RPMI1640 with Liberase TL (0.25 mg/ml, Roche) and DNase I (0.15 mg/ml, Sigma) at 37°C for 30 min. Tissue pieces were then mashed completely in Hank’s buffer (containing 1% FBS) and filtered through a 50-μm cell strainer to obtain single cell suspension of lamina propria.

### Primary CD4^+^ T Cells Culture

Mouse naïve CD4^+^ T cells (CD44^–^ CD62L^+^) were purified from spleens and lymph nodes of C57BL/6 mice. These cells were then cultured on 24-well plates pre-coated with 10 μg/ml plate-bound anti-CD3 (clone 145-2C11) and 5 μg/ml plate-bound anti-CD28 mAbs (clone 37.51) in complete RPMI (RPMI 1640 supplemented with 10% heat-inactivated FBS, 2mM-glutamine, 50 μM 2-ME, 100 U/ml penicillin and 100 μg/ml streptomycin) in various polarizing conditions: Th1, huIL-2 (20 U/ml, obtained from the BRB Preclinical Repository), IL-12 (3.4 ng/ml) plus anti-IL-4 (10 μg/ml, clone 11B11, from the BRB Preclinical Repository) and Th17, anti-IL-2Rα (10 μg/ml, clone PC61, American Type Culture Collection (ATCC), Manassas, VA, United States), IL-23(10 ng/ml), IL-6 (10 ng/ml), TGF-β1 (1 ng/ml), anti-IFN-γ (10 μg/ml, clone XMG 1.2), and anti-IL-4 (10 μg/ml, clone 11B11). At 48 h after the start of culture, cells were transferred to another plate, which was not coated with anti-CD3 or anti-CD28 Abs, with the original culture media (including polarizing cytokines and anti-cytokine Abs). Cells cultured in the Th1 condition were also supplemented with fresh human IL-2 (5 U/ml).

### *Ex vivo* Analysis of Immune Cells in Lymphoid Organs and Lamina Propria

Single cells were made from spleens, lymph nodes and lamina propria isolated from 6–8 week-old female wild type or BECN1 fl/fl mice. Cells were then stained with the following fluorescence-conjugated antibodies: CD45(30F11), CD4(GK1.5), CD8(53-6.7), CD44(IM7), CD62L(MEL-14), CD122(5H4), Ki67(SolA15), IL17(17B7), IFN-γ(XMG1.2) (eBioscience, Inc., San Diego, CA, United States) and p-S6(Cell Signaling). For intracellular cytokine staining, lymphocytes were stimulated with or without 10 ng/ml of PMA and 1 μg/ml of ionomycin (Sigma) for 4 h. Brefeldin A was added for the last 3 h at 10 μg/ml. Cells were transferred to a V-bottom plate, stained with anti-CD45, anti-CD4 and anti-CD8 Ab in Hank’s buffer (containing 1% FBS) and then fixed with 2% formaldehyde, which was followed by permeabilization with 0.5% saponin. The cells were subsequently stained with anti-Ki67 and anti-Foxp3 Ab or anti-IL-17 and anti-IFN-γ Ab (eBioscience, Inc., San Diego, CA, United States) or p-S6 Ab. Flow cytometric analysis was performed using a flow cytometer (BD Biosciences) and data were analyzed by using FlowJo (TreeStar).

### Statistical Analysis

Statistical analyses were performed using GraphPad Prism 5.0 (GraphPad Software, Inc., San Diego, CA, United States). Differences between experimental groups were analyzed by using student’s *t*-test or one-way ANOVA and all *P* values less than 0.05 were considered statically significant.

## Data Availability Statement

The raw data supporting the conclusions of this article will be made available by the authors, without undue reservation.

## Ethics Statement

The animal study was reviewed and approved by the University of Pittsburgh.

## Author Contributions

RX designed the experiments, performed the experiments, analyzed the data, and wrote the manuscript. MY analyzed the data and wrote the manuscript. XF, WD, XG, and GL performed the experiments and analyzed the data. SR performed the pathological analysis. XZ and JJ provided the key reagents and analyzed the data. BL designed the experiments, analyzed the data, and wrote the manuscript. All authors contributed to the article and approved the submitted version.

## Conflict of Interest

The authors declare that the research was conducted in the absence of any commercial or financial relationships that could be construed as a potential conflict of interest.
